# Study on Static Biomechanical Model of Whole Body Based on Virtual Human

**DOI:** 10.3390/s24206504

**Published:** 2024-10-10

**Authors:** Zheng Cheng, Bin Luo, Chuan Chen, Huajun Guo, Jiaju Wu, Dongyi Chen

**Affiliations:** 1 Institute of Computer Application, China Academy of Engineering Physics, Mianyang 621900, China; chengz@caep.cn (Z.C.); luobin1827@163.com (B.L.); chuanchen@caep.cn (C.C.); 2 Mobile Computing Center, University of Electronic Science and Technology of China, Chengdu 611731, China; fossetta@163.com (H.G.); dongyichen888@aliyun.com (D.C.)

**Keywords:** virtual person, biomechanical model, ergonomic analysis, sagittal plane static model

## Abstract

Material handling tasks often lead to skeletal injury of workers. The whole-body static biomechanical modeling method based on virtual humans is the theoretical basis for analyzing the human factor index in the lifting process. This paper focuses on the study of humans’ body static biomechanical model for virtual human ergonomics analysis: First, the whole-body static biomechanical model is constructed, which calculates the biomechanical data such as force and moment, average strength, and maximum hand load at human joints. Secondly, the prototype model test system is developed, and the real experiment environment is set up with the inertial motion capture system. Finally, the model reliability verification experiment and application simulation experiment are designed. The comparison results with the industrial ergonomic software show that the model is consistent with the output of the industrial ergonomic software, which proves the reliability of the model. The simulation results show that under the same load, the maximum joint load and the maximum hand load are strongly related to the working posture, and the working posture should be adjusted to adapt to the load. Upright or bent legs have less influence on the maximum load capacity of the hand. Lower hand load capacity is due to forearm extension, and the upper arm extension greatly reduces the load capacity of the hand. Compared with a one-handed load, the two-handed load has a greater load capacity.

## 1. Introduction

Ergonomics is an engineering and technical discipline that analyzes the impact of factors such as work environment and methods on human psychology, physiology, and other aspects by studying the operation of human–machine environment systems [1,2]. Virtual human efficiency analysis technology uses virtual humans instead of real workers [3,4] to conduct ergonomic analysis on workers. Due to its advantages such as low cost and reusability, biomechanical modeling technology based on virtual humans has rapidly developed with computer technology.

Musculoskeletal disease is the most common and serious occupational disease in traditional industrial manufacturing industries [5,6,7,8], usually caused by adverse factors such as excessive fatigue, excessive static load, poor posture, and repetitive operations [9]. It not only reduces the production efficiency and workability of workers [10] but also causes high medical costs, leading to national economic losses [11,12,13]. Research has shown that judging the difficulty of lifting solely based on material weight is far from enough [14]. The statistical results indicate that material lifting tasks often lead to worker injuries, with over 20% of compensation used to treat waist injuries [15,16], and up to 12% of work-related injuries caused by lifting human bodies [17,18]. Research has shown [19,20,21] that the L5/S1 lumbosacral joint between the fifth lumbar vertebra and pelvis, as well as the L4/L5 lumbar joint between the fourth and fifth lumbar vertebrae, are the most common sites of lumbar disc herniation. At the same time, the load capacity of lumbar vertebrae is correlated with age [22,23] and gender [24]. The load capacity of elderly people is poor, and the load capacity of women is about 5/6 of that of men. The above facts indicate that it is necessary to conduct mechanical analysis and prediction on the joints of the worker’s body, especially the spine, lumbar spine, and other parts, in lifting operations.

The development of computer technology has made it easier to use biomechanical data for developing analytical methods and studying human mechanics, promoting the development of human assessment models. According to different ways of obtaining human body information, human body evaluation models can be divided into three categories: (1) models based on manual observation, (2) a model based on electromyographic signals, and (3) a model based on anthropometry. Among them, models based on anthropometry can be divided into multirigid body mechanics models and finite element models.

(1) Models based on manual observation mainly include Rapid Upper Limb Assessment (RULA) [25] and Ovako Working Posture Analysis System (OWAS) [26]. These two methods directly observe human posture and joint angles, judge posture safety levels based on a scoring table, and provide corresponding suggestions. They can be used in scenarios that require visual judgment and do not require high safety accuracy. Although manual observation methods fully consider human posture and joint angles, their evaluation accuracy depends on the observer’s accuracy in posture judgment, which is subjective, lacks accuracy, lacks quantitative expression, and is not suitable for scenarios that require quantitative analysis of human mechanical responses.

(2) A model based on electromyographic signals: Dubois [27] and other researchers have shown that although there is a certain correlation between electromyographic indicators and muscle activity, it is difficult to represent or evaluate absolute muscle activity through electromyographic indicators due to individual differences in muscle activity, fatigue, and other factors [28,29,30]. The main research focuses on models that quantitatively study the comprehensive mechanical properties of muscles and local ligaments based on the internal dynamics of human movement [31,32,33,34,35,36], as well as hybrid musculoskeletal prediction models that have both motion prediction and muscle mechanics evaluation capabilities [37,38]. Due to the need to wear electromyography collection equipment to collect electromyographic signals, it can cause certain interference to the actual operation of workers and has certain limitations.

(3) Models based on anthropometry are mainly divided into finite element models and multirigid body models. Kong et al. [39] constructed a static sagittal plane biomechanical model using the finite element method. The predicted pressure values of the model were very close to those measured in vivo, but due to the limitations of the optimization method, there was uncertainty in its analysis of motion patterns. Domestic scholars [40,41,42] have studied the biomechanical response and injury mechanism in collision accidents. Based on adult lower limb CT image data, finite element models of the driver’s upper limb, pelvis, and lower limb were established, and the human response and injury application caused by collisions were studied, as well as gender correlation. In addition, some scholars [43] have established finite element models for human vibration response and lumbar load under long-term exposure to automotive vibration environments and studied the effects of vibration frequency and sitting posture on human biomechanical parameters. The advantage of the finite element modeling method is high precision, but it is difficult to model and has low computational efficiency. Through continuous development, finite element models have greatly improved in terms of mesh quantity, anatomical details, and material realism, but there is still significant room for development in terms of accuracy and anthropomorphism. The multirigid body model simplifies the human body into a hinged model composed of multiple rigid bodies [44] which can effectively simulate various complex connection problems of the human body. In the early stages, Morris et al. [45] first proposed a biomechanical model for predicting spinal biomechanics and intra-abdominal pressure. Pearson et al. [46] and Beijjani et al. [47] established a static model to describe the local tissue mechanical state of the human body. On this basis, Plagenhoef et al. [48] and Chaffin et al. [49,50,51,52] established a two-dimensional full-body biomechanical model of the human body. Schanne et al. [53] extended Chaffin’s two-dimensional biomechanical model to a three-dimensional model. Garg et al. [54] further added a hand maximum load prediction function to this three-dimensional biomechanical model. Baines [55] proposed a biomechanical model that can evaluate hand load based on a given body posture. The relevant research results have been applied to commercial ergonomic analysis software. There is relatively little research on multirigid body basic algorithm models in China. Wang et al. [56] used biomechanical methods to establish a biomechanical model for analyzing the force on the neck and waist under sitting conditions based on the characteristics of human body size and relevant parameters in China. The advantage of a multirigid body model is that it can demonstrate the anatomical characteristics of the human body and can be further applied to the study of biomechanical properties of the human body, such as joint load prediction. However, this model makes it difficult to reflect the stress and strain characteristics inside the tissue structure and is not suitable for scenarios that require a precise description of local tissue interactions.

In summary, the multirigid body model of anthropometry has more advantages in predicting and evaluating joint loads under lifting conditions in the industrial field due to its objectivity compared to observation models, absoluteness compared to electromyography models, and simplicity compared to finite element models. Therefore, this article focuses on the study of a full-body static biomechanical model for virtual artificial effects analysis, constructs a human body static biomechanical model, develops a testing and simulation platform, and simulates and verifies the ergonomic parameters of multiple human machines during lifting, handling, and other operations in industrial environments. The preliminary comparison and verification results with specialized human–machine ergonomic software show that the human joint reaction force and torque, average strength, maximum hand load, etc., output by the model are consistent and consistent with the output of specialized software, and maintain a low error rate. The purpose of this study is to predict multidimensional mechanical data of the human body using a human statics model, evaluate the suitability of work tasks, and improve the working methods of workers. The static biomechanical model of the human body uses biomechanical methods to establish the force and torque balance equations at various joints or waist positions of the human body connection system. The motion capture system obtains the position, posture, and other data of the real human body under common postures in the industry as input to predict the reaction force and reaction torque of various important joints in the human body connection system. Based on the predicted reaction force and reaction torque of important joints in the human body, we adjust and guide the work design to reduce human injuries.

## 2. Whole-Body Static Mechanical Model Framework

**Figure 1:**
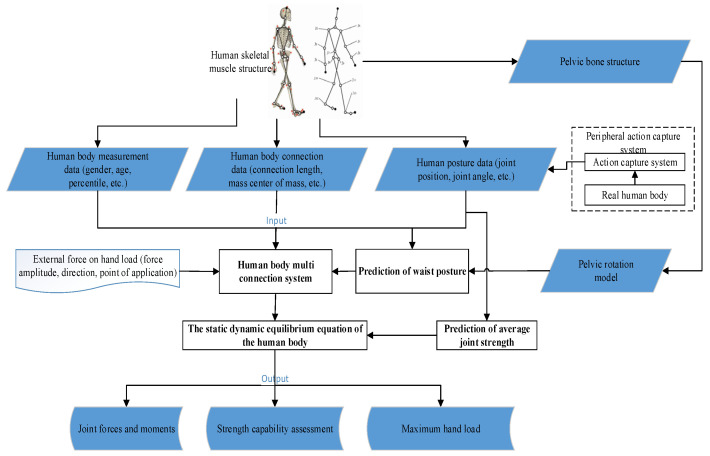
Schematic diagram of the algorithm framework for the full-body static mechanics model.

**Figure 3:**
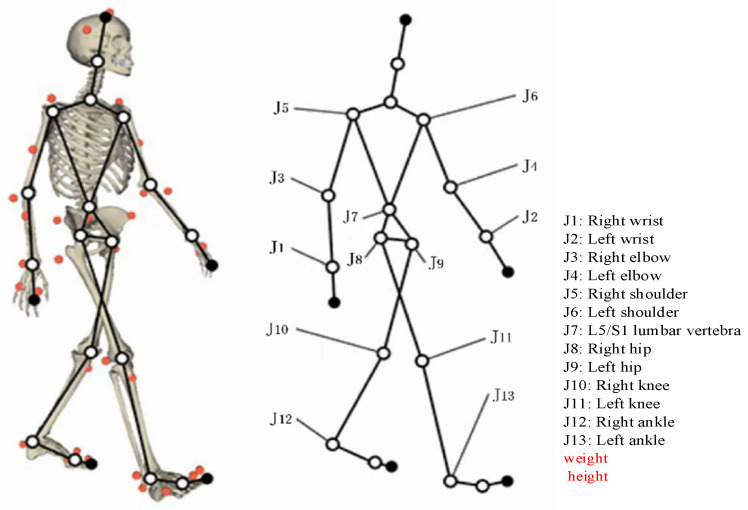
Joint mapping between human skeleton and whole-body static mechanics model.

The system framework for constructing a full-body static mechanical model is shown in Figure 1, which mainly consists of two parts: (1) an external motion capture system, mainly used to obtain data on the position, posture, and other aspects of the real human body; (2) the whole-body static biomechanical model is mainly composed of a multiconnected human body system, a pelvic rotation model, and dynamic equilibrium equations. Among them, the human multiconnection system is based on the human skeletal muscle structure to construct a connection motion system, the pelvic rotation model predicts the spatial position of the lumbar spine based on the pelvic structure motion, and the dynamic balance equation uses biomechanical methods to establish the force and torque balance equations at each joint or waist position of the connection system, predicts the reaction force and reaction torque of important joints in the human connection system, and combines the calculated average joint strength to predict and evaluate the maximum load force that the human hand can bear when performing lifting tasks. The input data of the whole-body static biomechanical model include (a) human body measurements data such as gender, age, and percentile; (b) human body connection data such as length, mass, and center of mass; (c) human pose data such as joint spatial position and joint angle obtained from the peripheral action capture system; (d) the amplitude, direction, and point of application of external force on the hand load. The output data of the model include the force and torque of human joints, strength capability evaluation, etc. The J_1_–J_13_ is described in Section 3.1 and Figure 3.

**Figure 2:**
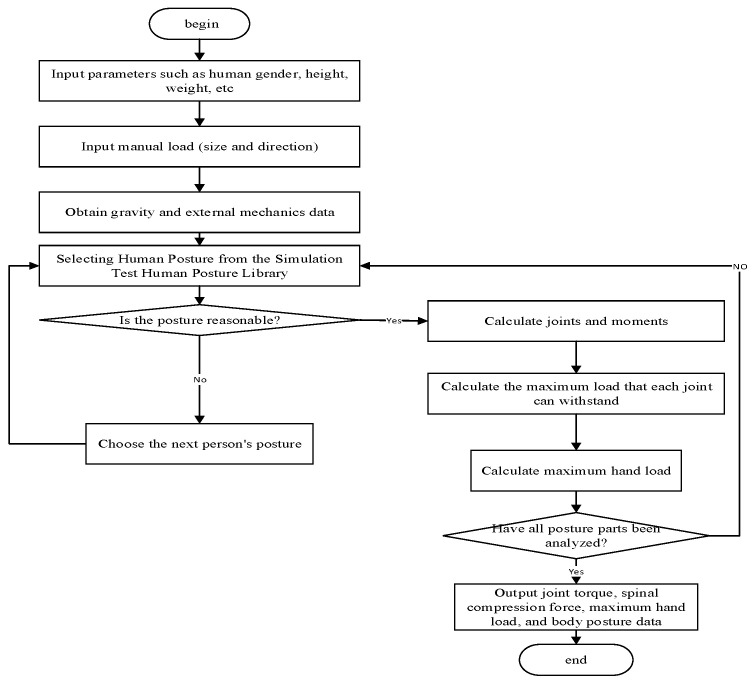
Schematic diagram of the calculation process for the full-body static mechanical model.

The calculation process of the whole-body static mechanics model is shown in Figure 2, which mainly includes the following steps. The first step is to input human body data such as gender, height, and weight; Step 2: input the hand load; Step 3: input other external mechanical data including gravity; Step 4: input the human posture data collected by the motion capture system to determine whether the posture is reasonable (whether it is achievable, whether it exceeds the limits of each joint of the human body). If it is reasonable, calculate the mechanical data at each joint and the maximum hand load corresponding to that posture. If it is unreasonable, choose the next human posture. Finally, check if all the postures included in the posture database have been analyzed. If not, select the next human posture and repeat the fourth step. If yes, output the relevant prediction data and end.

## 3. Virtual Human Whole-Body Static Biomechanical Model

### 3.1. Human Body Connection System and Waist Posture Calculation

The human motion system is an organic and complex whole composed of 206 bones, over 600 muscles, and countless neurons that command the movement of these bones and muscles. According to the motion laws of human joints and bones, a virtual human model can be simplified as a system composed of connections and joints, where connections are defined as rigid bones between two important joints in the human body, and adjacent connections are hinged by joints. This article describes a virtual human connectivity system consisting of 12 body connections and 13 body joints, as shown in Figure 3. The 13 joints include symmetrical wrists, elbows, shoulders, hips, knees, ankles, and lumbar vertebrae. The 12 connections include 2 hands, 2 forearms, 2 large arms, 2 thighs, 2 calves, 1 shoulder lumbar spine, and 1 lumbar spine hip joint. The biological parameters such as length, mass, center of mass position, and range of motion of each joint for each connection are referred to in reference [51].

The lumbar spine (J7) joint is prone to injury or pathological changes, and its spatial position and angle vary with the pelvic rotation caused by human pitching motion. The pelvis is a combination of bones located below the lumbar vertebrae of the body, including the lumbosacral joint and hip joint. In an upright position, the pelvis is in a relatively vertical position, but in a forward leaning or pitching position of the human torso, the pelvis will rotate around its center of rotation. In the human connectivity system, the pelvis is defined as a connection between the double hip and L5/S1 lumbosacral joints; therefore, the spatial position of the lumbar spine must be located by calculating the direction of the pelvis through pelvic rotation. This article uses the degradation equation based on knee and torso angles proposed in reference [57] to predict the pelvic rotation angle when the human body is upright and the rotation angle of L5 relative to the sacrum. According to reference [51], the spatial position of L5/S1 is calculated from the hip center spatial position and pelvic rotation angle.

### 3.2. Equilibrium Equation for Multiple Connections in the Sagittal Plane of the Human Body

In the construction of a multiconnected system, the joint forces and torques exerted by the human body’s load and hand loads on the sagittal plane have a serious impact on joint injuries, especially lumbar spine injuries. This article uses the multiconnection static equilibrium equation to calculate the reaction force and torque of human joints in the sagittal plane of the human body. On the sagittal plane of the human body, the external force of the human hand and the gravity of the body connection satisfy the force balance equation in the vertical direction of the connection system’s various joint positions, that is, the reaction force balance equation at each joint can be described as Formulas (1) and (2).

(1) $$\sum R_{j}=0$$

(2) $$R_{j}=R_{j-1}+W_{L}$$

Among them, $R_{j}$ is the reaction force at joint j, $R_{j-1}$ is the reaction force at the previous joint j − 1, and $W_{L}$ is the weight of the L connection.

The torque balance equation at each joint of the connecting system can be described as Formulas (3) and (4).

(3) $$\sum M_{j}=0$$

(4) $$M_{j}=M_{j-1}+\left({CM}_{Lj}cos\left(\theta_{j}\right)W_{L}\right)+\left(L_{j,j-1}cos\left(\theta_{j}\right)R_{j-1}\right)$$

Among them, $M_{j}$ is the reaction torque at joint j, ${CM}_{Lj}$ is the distance from joint j to the center of mass connecting L, $\theta_{j}$ is the angle between the L connecting line at joint j and the horizontal line, and $L_{j,j-1}$ is the length of the connection from joint j to joint j − 1.

Each joint of the human body satisfies the law of mechanical equilibrium. Based on the above reaction force and moment balance equations, the joints in the static mechanical model of the whole body are analyzed separately. The calculation method for body joints with sagittal symmetry is the same, starting from the upper limb of the human body, connecting adjacent joints, and finally reaching the lower leg; the reaction force and moment at the wrist, elbow, shoulder, L5/S1 lumbosacral, hip, knee, and ankle joints of the human body are calculated separately.

### 3.3. Average Strength and Maximum Hand Load

Muscle strength refers to the maximum force that joint muscles can withstand in a certain posture, also known as maximum autonomous force application. The force exerted when the body connection is restricted and no movement occurs is called static strength. The average strength is defined as the maximum torque strength that a joint in the human body’s connecting system can withstand, meeting the following limitations like formula (5).

(5) $$-S_{j}<M_{Lj}<S_{j}$$

Among them, $M_{Lj}$ is the torque at joint j caused by the external force L on the hand and the weight of the body connection. $S_{j}$ is the average strength of the torque generated by the muscles at joint j. When the torque of the human joint is outside its corresponding average strength range, it indicates that the joint will be damaged. The average strength of each joint in the human body is closely related to the angle at that joint, and its calculation method can be referred to in reference [51].

The average strength of each joint in the human body is closely related to the angle at that joint and the gender of the body. Therefore, different postures and genders correspond to different values of average joint strength, which can be regarded as a function with joint angle and gender as independent variables.

Let the human elbow angle $\theta_{E}$ be the inner angle between the upper arm and the lower arm. The angle between the upper arm and the human torso is $\theta_{s}$. The angle between the shoulder L5/S1 lumbar joint line and the knee hip joint line is $\theta_{T}$. The angle between the shoulder hip joint line and the thigh is $\theta_{H}$. The angle between the thigh and calf is $\theta_{K}$. The angle between the calf and the horizontal plane is $\theta_{A}$. The gender adjustment coefficient is G. According to reference [51], the calculation method for the average intensity of joint movements in the human wrist, elbow, shoulder, L5/S1 lumbar spine, hip, knee, ankle, and other joints can be obtained.

The calculation method for the average strength of elbow bending is shown in the Formula (6).

(6) $$S_{E}=\left(336.29+1.544\theta_{E}-0.0085{\theta_{E}}^{2}-0.5\theta_{S}\right)*G$$

The calculation method for the average strength of elbow extension is shown in Formula (7).

(7) $$-S_{E}=\left(264.153-0.575\theta_{E}-0.425\theta_{E}\right)*G$$

The calculation method for the average strength of shoulder bending is shown in Formula (8).

(8) $$S_{s}=\left(227.338+0.525\theta_{E}-0.296\theta_{s}\right)*G$$

The calculation method for the average strength of shoulder extension is shown in Formula (9).

(9) $${-S}_{s}=\left(204.562-0.099\theta_{s}\right)*G$$

The calculation method for the average strength of trunk bending is shown in Formula (10).

(10) $${-S}_{T}=\left(17.17\theta_{T}-0.0796{\theta_{T}}^{2}\right)*G$$

The calculation method for the average strength of trunk extension is shown in Formula (11).

(11) $$S_{T}=\left(3894-13.9\theta_{T}\right)*G$$

The calculation method for the average strength of hip curvature is shown in Formula (12).

(12) $$S_{H}=\left(-820.21+34.29\theta_{H}-0.11426{\theta_{H}}^{2}\right)*G$$

The calculation method for the average strength of knee bending is shown in Formula (13).

(13) $${-S}_{K}=\left(-94.437+6.3672\theta_{K}\right)*G$$

The calculation method for the average strength of knee extension is shown in Formula (14).

(14) $$S_{K}=\left(1091.9-0.0996\theta_{K}+0.17308{\theta_{K}}^{2}-0.00097{\theta_{K}}^{3}\right)*G$$

The calculation method for the average strength of ankle extension is shown in Formula (15).

(15) $${-S}_{A}=\left(3356.8-18.4\theta_{A}\right)*G$$

When the human body is performing tasks such as hand lifting, pushing, and pulling objects, the greater the load on the hand, the greater the force and torque intensity on the human joints. When the joint torque intensity is equal to its average strength, the hand bears the maximum load gravity. When the hand load exceeds the maximum load, the joint will suffer damage. The maximum load on this hand represents the maximum strength capacity of the human body, and it is necessary to predict and calculate the maximum load on this hand. According to the torque balance equation, when the hand increases the load, the joint torque caused by the load will increase proportionally to the torque arm of the load to the joint. Therefore, the torque at the joints is composed of two parts: the torque caused by the weight of the human body and the torque caused by the load on the hands, which can be expressed as follows:

(16) $$M_{j,L}=M_{j,L=0}+L_{jh}W_{load}$$

Among them, $M_{j,L}$ represents the moment at joint j when there is hand load, $M_{j,L=0}$ represents the moment at joint j caused solely by human weight without hand load, and $L_{jh}$ is the moment arm from the hand load position to joint j.

If the torque at joint j is set to be equal to its average torque strength $S_{j}$, the maximum load on the hand when joint j is not damaged can be calculated as per Formula (17).

(17) $$W_{max:j}=\frac{\left(S_{j}-M_{j,L=0}\right)}{L_{jh}}$$

To ensure that all joints of the human body are not damaged under a certain lifting condition, the maximum load on the human hand must not exceed the minimum load value that can be borne by all joints. Therefore, the maximum load on the human hand is equal to the minimum value of the maximum load on the hand corresponding to all joints, which is like Formula (18).

(18) $$W_{max:h}=Min\left(W_{max:1},W_{max:2},\ldots \ldots ,W_{max:j},W_{max:j+1},\ldots \ldots \right)$$

## 4. Algorithm Simulation and Validation

### 4.1. Simulation Testing Platform

**Figure 4:**
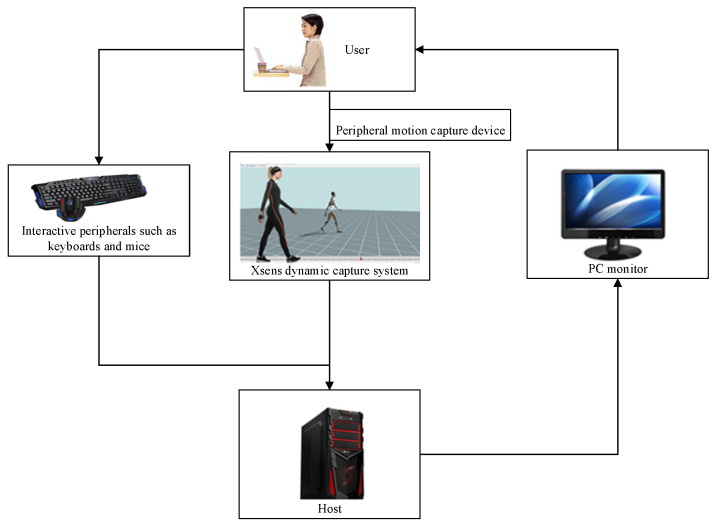
Verification environment for full-body static mechanics model.

The experimental platform used for testing and verifying the whole-body static mechanical model consists of an external motion capture system and an ergonomic component (ERG) prototype system for ergonomic analysis and evaluation. The peripheral motion capture system uses the Xsens MVN inertial motion capture system to obtain real human joint positions, body posture, and other data. ERG is developed based on the Visual Studio platform, which obtains human spatial position and posture data from an external motion capture system, inputs it into a full-body static mechanical model, and performs ergonomic evaluations such as static strength prediction and waist force prediction on virtual humans. It calculates biomechanical indicators such as joint reaction force and torque, average joint strength, and maximum hand load during lifting operations. The experimental environment for algorithm simulation verification is shown in Figure 4.

The test prototype system ERG is developed based on Visual Studio 2005 and the CATIA CAA (Component Application Architecture) secondary development platform, running on Windows 10 or Windows 11 operating systems. CAA is a tool provided by CATIA for secondary development and extension. Users can communicate with CATIA by installing the RADE (Rapid Application Development Environment) module and using the interface provided by CAA to call the underlying functions of CATIA, obtain the core data of the object model, edit the user graphical interface, and achieve more functions required by users. The test prototype system ERG V1.0.0 software is similar to 3DSSPP and JACK V8.01 software, and the model is effective. The core algorithm of JACK software is the same as 3DSSPP V7.1.3 software.

The input data for the ERG software of the test prototype system include (1) human body measurements data such as gender, age, height, and weight; (2) human body posture data such as joint spatial position and joint angle; (3) the amplitude, direction, and point of application of external force on the hand load. The output interface of the test prototype system includes two types: output data and output graphics. The output data include human joint forces and torques, strength capacity evaluation, maximum hand load, etc.

The ERG system developed in this article uses the Xsens MVN inertial motion capture system to verify the virtual human and static algorithm models with real human learning situations. When the verification of the virtual human and static model algorithms is completed and the system is in normal use, human body data can be directly input, and the verified virtual human can be called to perform static prediction, calculate mechanical data, and adjust the work plan and steps. The ERG prototype system obtains spatial position data of the wrist, elbow, shoulder, torso, hip, knee, and ankle joints of the human body from the peripheral motion capture system. The obtained data are input into the static strength model of the human body to perform efficiency evaluations such as static strength prediction and waist force prediction on the virtual human. It calculates relevant data such as joint angle, joint average strength, joint reaction force, and torque when lifting heavy objects. Researchers can use input devices such as mice and keyboards to input operation instructions and send commands to the ERG system to perform full-body static mechanical force analysis on virtual human bodies in the scene, providing users with an interactive operating experience.

### 4.2. Simulation Testing Human Posture Library

A questionnaire survey was conducted on 20 transportation workers from a cargo transportation company in Sichuan Province. Six postures were selected, including upright posture, right forearm extension, double arm abduction, right upper arm extension, squatting upper arm extension, and standing hands extension, to construct a human posture database for verification and application of simulation experiments. The testers wear Xsens MVN motion capture clothing and perform lifting operations on heavy objects in the selected posture. They use professional software provided by the Xsens MVN motion capture system to determine the three-dimensional coordinates, joint angles, and other position and posture data of the human body connection and joints in different postures. We import the above data into the ERG prototype system, and the interface will display them, as shown in Table 1.

### 4.3. Reliability Verification Experiment

We select an industrial-specific ergonomics software and test prototype system ERG to compare the predicted output of joint average strength, joint torque, and spinal compression force, to measure the accuracy of ERG software output.

The reliability verification of the model includes three steps: (1) Determine the parameters of the virtual human for simulation verification in ERG and specialized software, including gender, height, weight, etc., and create the virtual human; (2) select the poses in the pose library one by one, and import them into ERG and specialized software to calculate them separately; (3) change the virtual human hand load in different postures, record the output data corresponding to various indicators of ERG and specialized software, and compare and analyze the output data.

In the experiment, the dimensions of the virtual human used in the specialized software and ERG software are shown in Table 2. The specialized software includes SAMMIE developed by the University of Nottingham, 3DSSPP developed by the University of Michigan, JACK developed by the University of Pennsylvania, RAMSIS developed by the Technical University of Munich and Human Solutions, ANYBODY widely used in recent years, SAFEWORK developed by Canadian researchers, etc. The JACK kernel adopts the same algorithm as 3DSSPP, which is proposed by the Center for Human Engineering at the University of Michigan in the United States. Due to the classic and widely used static force prediction algorithm proposed by the Human Center of the University of Michigan in the United States, this article selects software related to this algorithm for comparison.

The reliability and accuracy of the model are quantitatively described using error degrees. The calculation method is (A − E)/(E/100%), where A represents the measured value, which is the output data of ERG, and E represents the standard value, which is the output data of specialized software. A positive error indicates that the measured value is greater than the standard value, while a negative error indicates that the measured value is less than the standard value.

#### 4.3.1. Average Joint Strength

**Figure 5:**
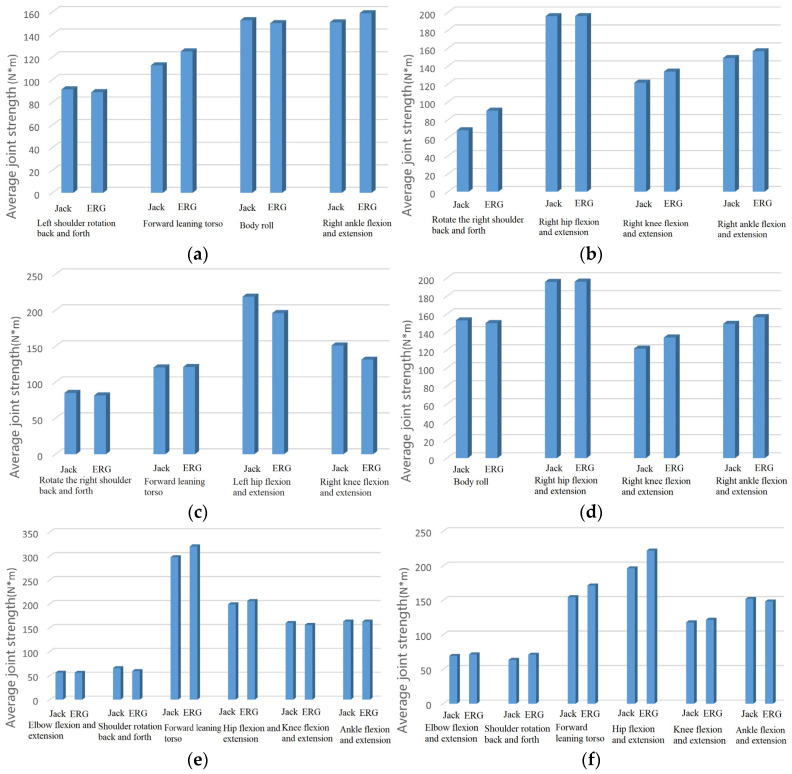
Average joint strength output data of ERG and specialized software under different postures. (**a**) Upright posture; (**b**) right forearm extension posture; (**c**) arm abduction posture; (**d**) right upper arm forward extension posture; (**e**) squat down and extend the upper arm forward; (**f**) standing with hands extended forward posture.

Verification method: For the six postures shown in Table 1, we record the average strength data of each joint of the virtual human output by ERG and specialized software for comparison and analysis. Figure 5 shows the average strength of each joint calculated by ERG and specialized software Jack under six different postures, and Table 3 shows the corresponding joint average strength error.

From Figure 5 and Table 3, it can be observed that (1) the average joint strength output of ERG and specialized software is consistent under the six postures included in the simulation test human posture library, that is, the output data are the same; (2) the average joint strength error output by ERG and specialized software remains at a relatively low level.

It can be seen that the whole-body static mechanics model can accurately predict the average strength of each joint in the worker’s body.

#### 4.3.2. Joint Torque

**Figure 6:**
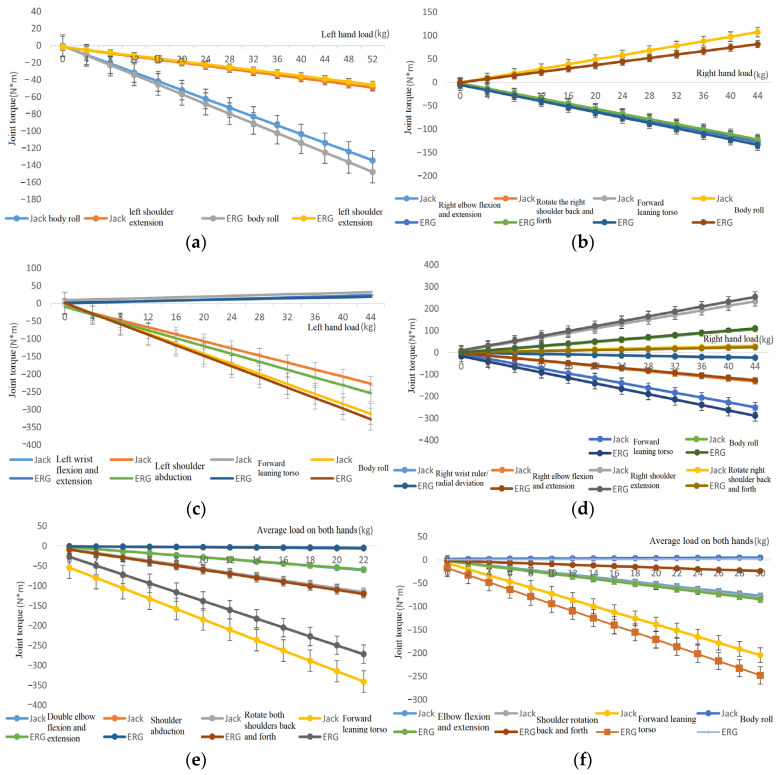
Joint torque output data of ERG and specialized software under different postures. (**a**) Upright posture; (**b**) right forearm extension posture; (**c**) arm abduction posture; (**d**) right upper arm forward extension posture; (**e**) squat down and extend the upper arm forward; (**f**) standing with hands extended forward posture.

Verification method: Starting from 0, we gradually increase the hand load for the six postures shown in Table 1, record the joint torque changes in various directions of the virtual human in ERG and specialized software, and compare and analyze the output data. Figure 6 shows the changes in joint torque output for six different postures calculated by ERG and specialized software. Table 4 shows the load changes and corresponding calculated joint torque errors for each posture.

From Figure 6 and Table 4, it can be observed that (1) the joint torque output and its changes corresponding to ERG and specialized software are consistent under the six postures included in the simulation test human posture library, that is, the output data curves basically overlap, while increasing or decreasing, showing good fitting. (2) The average error of joint torque output by ERG and specialized software remains at a low level, with good accuracy.

It can be seen that the whole-body static mechanics model can accurately predict the joint torque data of workers.

#### 4.3.3. Spinal Compression Force

**Figure 7:**
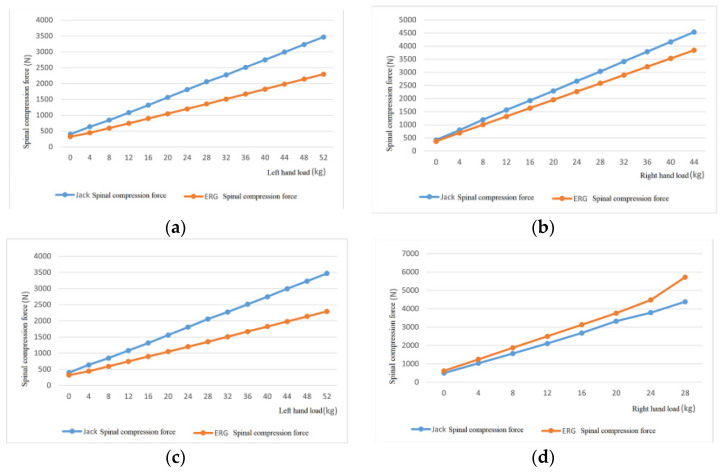

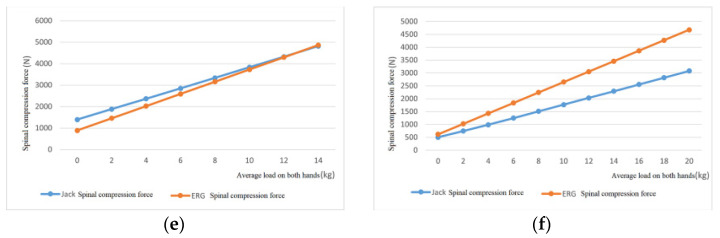
Spinal compression force output data of ERG and specialized software under different postures. (**a**) Upright posture; (**b**) right forearm extension posture; (**c**) arm abduction posture; (**d**) right upper arm forward extension posture; (**e**) squat down and extend the upper arm forward; (**f**) standing with hands extended forward posture.

Verification method: For the six postures shown in Table 1, we select the same load conditions as Table 4, gradually increase the hand load from 0, record the changes in spinal compression force at the lumbosacral joint of virtual human L5/S1 in ERG and specialized software, and compare and analyze the output data. Figure 7 shows the changes in spinal compression force output for six different postures calculated by ERG and specialized software.

From Figure 7, it can be observed that (1) under six different postures, the output and variation of spinal compression force corresponding to ERG and specialized software are consistent, that is, the output data curves overlap while increasing or decreasing, showing good fitting. (2) The error level of spinal compression force output by ERG and specialized software remains at a relatively low level. Except for the spinal compression force error of squatting upper arm forward extension posture, which gradually decreases with increasing load, the error level of spinal compression force is small when the load is small in the other five postures. As the load increases, the error level gradually increases.

It can be seen that the whole-body static mechanics model can accurately predict the spinal compression force data of workers.

Under no-load or low-load conditions, the output joint torque and spinal compression force data of ERG software have considerable effectiveness, maintaining a relatively low level of relative error compared to the output of Jack software.

As the hand load increases, the joint torque error in the same direction output by the two software gradually increases, and the overall error increase is relatively small. When the hand load exceeds a certain level, the relative error of the predicted spinal compression force output by ERG is smaller than that of Jack’s predicted value. The spinal compression force value output by ERG is more accurate than Jack’s.

The trend of joint torque and spinal compression force data output by ERG software is consistent with Jack, that is, both increase and decrease simultaneously, and the fluctuation situation remains consistent. The average intensity data output by ERG software can always fit Jack’s output data well. The main reason for the error in the output of the two is the system error caused by different parameter choices such as human body measurement parameters and human body connection parameters, as well as the calculation bias inherent in the algorithm itself.

### 4.4. Application Simulation Experiments

The application simulation experiment mainly predicts and evaluates the maximum hand load of virtual humans in different postures. The virtual human parameters created in the experiment are the same as those in Table 2. The experimental steps are as follows: (1) Select the virtual human pose from the pose library in Table 1; (2) increase the hand load using the same method as shown in Table 3, and record the joint torque output data in each direction; (3) corresponding to the output torque of each joint to the average strength of the joint in that posture, calculate the maximum hand load that each joint can withstand; (4) the minimum value of the maximum load on the hands of each joint is taken as the predicted maximum load on the hands in that posture.

**Figure 8:**
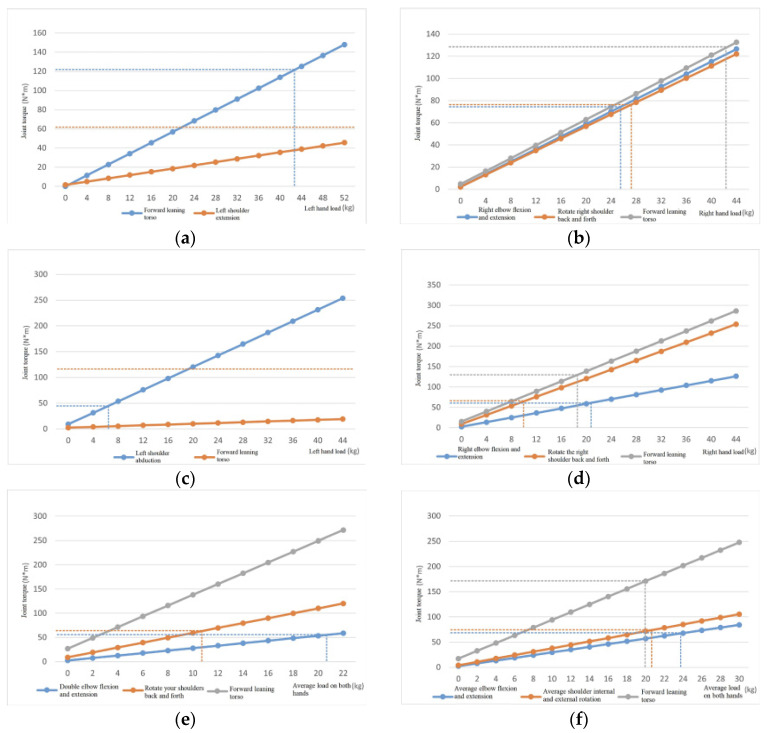
Simulation of maximum hand load prediction for ERG under different postures. (**a**) Upright posture; (**b**) right forearm extension posture; (**c**) arm abduction posture; (**d**) right upper arm forward extension posture; (**e**) squat down and extend the upper arm forward; (**f**) standing with hands extended forward posture.

The corresponding relationship between the maximum load on the hand and the changes in joint torque and average joint strength under six different postures is shown in Figure 8. The colored solid lines in the figure represent the changes in joint torque in each direction with the change in hand load. The horizontal dashed lines of the corresponding colors represent the average joint strength in that direction, and the vertical dashed lines represent the size of the hand load corresponding to the maximum torque that the joint can bear. The maximum torque that the joint can bear and the joint torque generated by the weight of the human body are combined to calculate the maximum load on the hand corresponding to the joint. In the same posture, the maximum load on the human hand is the minimum value among the maximum loads corresponding to each joint. Table 5 shows the calculated maximum load on the human hand and the corresponding joint torque directions for reaching the maximum load.

From Table 5, it can be observed that (1) the maximum hand load values corresponding to different postures are different, and there are significant differences; (2) the shoulder rotation direction is most likely to reach maximum load, followed by the knee flexion and extension direction, and, finally, the ankle flexion and extension direction; (3) the impact of upright and bent leg states on the predicted maximum hand load is relatively small; extending the forearm forward can lead to a decrease in hand load capacity; extending the upper arm forward can significantly reduce the hand’s load capacity; (4) compared to single-handed loads, two-handed loads can withstand greater hand loads.

From this, it can be seen that in the process of material lifting, it is advisable to choose a posture with the arms (including forearms and forearms) hanging down to lift heavy objects and to choose double arm lifting instead of single arm lifting as much as possible. In addition, we do not attempt to lift heavy objects that exceed the predicted maximum load on the hands to avoid irreversible harm to the body and effectively reduce the probability of muscle and bone damage to workers.

## 5. Conclusions

The analysis of human factors indicators based on virtual human static biomechanical models is a key technology in the application of occupational biomechanics in industrial lifting operations. In response to the situation where material lifting tasks often cause bone damage to workers, this article focuses on the research of human static biomechanical models for virtual artificial efficiency analysis, aiming to improve the work experience of workers. The main work content and specific contributions are summarized as follows:

(1) The overall design scheme for the research on the static mechanical model of a virtual human’s entire body in the homework scenario was completed. The article constructed a static biomechanical model framework for the whole body, established a virtual human connection system, clarified relevant human measurement parameters and their acquisition methods, and predicted the position and posture of the L5/S1 lumbosacral joint.

(2) This paper established a static mechanical model of the entire virtual human body using the multiconnected balance equation of the human body. Starting from the upper limbs of the human body, the mechanical data corresponding to each joint included in the virtual human body segment model were calculated separately. We calculated the maximum load on the virtual human hand corresponding to the postures in the common human posture database for transportation, based on the average strength of the joints.

(3) We developed a static model testing prototype system (ergonomic component; ERG), combined with an inertial motion capture system to establish a real-person experimental environment, and built a simulation testing platform. Regarding the virtual human skeletal system structure for static mechanical analysis of the entire body in lifting operation scenarios, we clarified the human measurement parameters and their acquisition methods required for analysis, and combined inertial motion capture devices to achieve the mapping from real human to virtual human.

(4) We designed a reliability verification experiment and application simulation experiment for a full-body static biomechanical model based on a simulated human posture library. We completed the reliability verification experiment and model application simulation of a virtual human full-body static biomechanical model based on common human posture libraries in the transportation industry. The reliability of the whole-body static mechanics model was preliminarily tested and evaluated using human body data in the posture library, demonstrating the effectiveness of the model. The model was applied to predict the maximum hand load of workers in lifting operation scenarios in the industry, and, based on the prediction results, recommendations were given for lifting operations for transportation industry practitioners.

Based on the above research results, this article can further conduct research in the following areas:

(1) The reliability verification of the model in this article selected the industrial-specific human–machine ergonomics software Jack for output comparison. The verification of joint torque, spinal compression force, and average joint strength all showed that the model in this study has considerable effectiveness, but there are still some system errors or calculation deviations. In future work, the calculation accuracy of the model can be further improved.

(2) The whole-body static mechanical model in this article studies the mechanical characteristics of workers during their work process in a static state, which can serve as a theoretical basis for dynamic analysis. In subsequent work, the momentum factor during workers’ work process can be considered and dynamic analysis can be added. Combining this with a real-time motion capture system will achieve dynamic mechanical characteristic analysis of workers during the operation process.

(3) In this article, biomechanical indicators such as joint force and torque, average joint strength, and maximum hand load of virtual humans were studied. Subsequent research can promote the application of virtual human models in other human–machine efficiency analysis such as human fatigue recovery analyses, quantitatively analyze worker fatigue in the transportation industry, and provide more comprehensive work guidance and suggestions.

## Figures and Tables

**Table 1:** Software simulation test human posture library.

Erect	Right Forearm Extension	Arm Abduction	Right Upper Arm Extension	Squat down and Extend the Upper Arm Forward	Standing with Hands Extended Forward
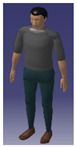	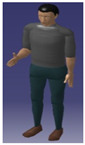	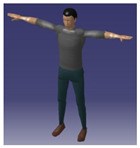	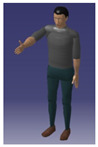	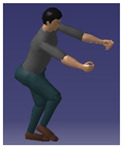	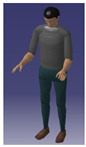

**Table 2:** Virtual human parameters in specialized software and ERG software.

Software	Virtual Human Height (cm)	Virtual Human Weight (kg)
Certain specialized software	175.49	77.69
Test prototype system ERG	178.34	61.84

**Table 3:** Average joint strength output error of ERG and specialized software under different postures.

Attitude	Erect	Right Forearm Extension	Arm Abduction	Right Upper Arm Extension	Squat down and Extend the Upper Arm Forward	Standing with Hands Extended Forward
Average error degree (%)	4.80	4.67	5.04	4.01	3.91	6.77

**Table 4:** Load conditions and average joint torque output error of ERG and specialized software under different postures.

Attitude	Erect	Right Forearm Extension	Arm Abduction	Right Upper Arm Extension	Squat down and Extend the Upper Arm Forward	Standing with Hands Extended Forward
Load situation (kg)	Left hand load 0–52	Right hand load 0–44	Left hand load 0–44	Right hand load 0–44	Hand load 0–30	Left hand load 0–30
Average error degree (%)	3.14	1.39	4.83	3.07	1.70	7.62

**Table 5:** Predicted maximum hand load and corresponding joint torque direction under different postures.

Attitude	Erect	Right Forearm Extension	Arm Abduction	Right Upper Arm Extension	Squat down and Extend the Upper Arm Forward	Standing with Hands Extended Forward
Maximum hand load (N)	1439.69	269.70	828.71	83.66	85.95	180.05
Joint torque direction	Left knee flexion and extension	Right shoulder pronation	Ankle flexion and extension	Right shoulder pronation	Right knee flexion and extension	Right shoulder pronation

## Data Availability

The data can be accessed from this manuscript.
